# Rationally Designed Molecularly Imprinted Polymer Electrochemical Biosensor with Graphene Oxide Interface for Selective Detection of Matrix Metalloproteinase-8 (MMP-8)

**DOI:** 10.3390/bios15100671

**Published:** 2025-10-04

**Authors:** Jae Won Lee, Rowoon Park, Sangheon Jeon, Sung Hyun Kim, Young Woo Kwon, Dong-Wook Han, Suck Won Hong

**Affiliations:** 1 Department of Cogno-Mechatronics Engineering, College of Nanoscience and Nanotechnology, Pusan National University, Busan 46241, Republic of Korea; jaewon03135@gmail.com (J.W.L.); rowoon.p153@gmail.com (R.P.); sangheon.jn@gmail.com (S.J.); nanohan@pusan.ac.kr (D.-W.H.); 2 Engineering Research Center for Color-Modulated Extra-Sensory Perception Technology, Pusan National University, Busan 46241, Republic of Korea; ksh1332@pusan.ac.kr (S.H.K.); ywkwon@pusan.ac.kr (Y.W.K.)

**Keywords:** molecularly imprinted polymers, matrix metalloproteinase-8, cytokine, electrochemical impedance spectroscopy, point-of-care testing

## Abstract

Molecularly imprinted polymer (MIP) biosensors offer an attractive strategy for selective biomolecule detection, yet imprinting proteins with structural fidelity remains a major challenge. In this work, we present a rationally designed electrochemical biosensor for matrix metal-loproteinase-8 (MMP-8), a key salivary biomarker of periodontal disease. By integrating graphene oxide (GO) with electropolymerized poly(eriochrome black T, EBT) films on screen-printed carbon electrodes, the partially reduced GO interface enhanced electrical conductivity and facilitated the formation of well-defined poly(EBT) films with re-designed polymerization route, while template extraction generated artificial antibody-like sites capable of specific protein binding. The MIP-based electrodes were comprehensively validated through morphological, spectroscopic, and electrochemical analyses, demonstrating stable and selective recognition of MMP-8 against structurally similar interferents. Complementary density functional theory (DFT) modeling revealed energetically favorable interactions between the EBT monomer and catalytic residues of MMP-8, providing molecular-level insights into imprinting specificity. These experimental and computational findings highlight the importance of rational monomer selection and nanomaterial-assisted polymerization in achieving selective protein imprinting. This work presents a systematic approach that integrates electrochemical engineering, nanomaterial interfaces, and computational validation to address long-standing challenges in protein-based MIP biosensors. By bridging molecular design with practical sensing performance, this study advances the translational potential of MIP-based electrochemical biosensors for point-of-care applications.

## 1. Introduction

Periodontitis, a prevalent inflammatory condition affecting the periodontal tissues, represents one of the most significant challenges in current dental medicine [1]. This progressive disease threatens oral health through potential tooth loss and has significant systemic consequences, particularly in relation to cardiovascular diseases and diabetes mellitus [2]. While traditional diagnostic approaches remain largely dependent on clinical expertise and time-intensive procedures [3], emerging research has integrated the critical need for more efficient and objective diagnostic methodologies [4]. The growing recognition of the bidirectional relationship between periodontal health and systemic conditions has intensified the demand for innovative diagnostic tools that can facilitate preventive interventions [2,4]. Early detection and diagnosis of periodontitis can significantly improve treatment outcomes, enhance patient compliance, and reduce the associated economic burden. On the other hand, current diagnostic protocols primarily rely on radiological observations and clinical interpretations rather than quantitative analysis of underlying biological markers [3,4]. These limitations on the molecular detection have raised considerable interest in the identification and validation of specific biomarkers in oral fluids, particularly for their potential application in initial diagnosis and longitudinal monitoring of periodontal disease progression [5,6].

In this context, saliva has emerged as an ideal diagnostic medium, offering rich clinical information through non-invasive collection methods that require minimal technical expertise [6]. Among the various salivary biomarkers, matrix metalloproteinase-8 (MMP-8) has demonstrated particular promise in periodontitis disease differentiation [7,8,9]. This collagen-degrading enzyme, integral to connective tissue metabolism, has shown consistent correlation between its elevated levels in gingival crevicular fluid (GCF) and saliva with periodontal inflammation and tissue destruction [9]. Consequently, salivary MMP-8 detection can be a valuable diagnostic indicator for the presence and progression of periodontitis. Recent point-of-care (POC) diagnostic technologies primarily utilize lateral flow immunochromatography, with some applications incorporating enzyme-linked immunosorbent assay (ELISA) methodology for active MMP-8 detection in oral specimens [8,9]. While prototype POC devices measuring alternative biomarkers such as neutrophil elastase in GCF and salivary C-reactive protein have been developed, comprehensive clinical validation data remain limited [10,11,12]. Thus, developing a viable methodology for the rapid, sensitive, and precise detection of the biomarkers can be critical in diagnostic medicine of periodontitis. Among other promising technologies, electrochemical biosensors have facilitated the transduction of biomarker-specific chemical signatures from minimal volumes of biofluids (i.e., saliva, tears, and blood) into concentration-dependent electrical signals [13]. Some sophisticated biosensor systems demonstrated exceptional potential for addressing diverse diagnostic challenges, especially in POC applications.

Within advanced biosensing platforms, molecularly imprinted polymers (MIPs) have emerged as a transformative technology, offering unparalleled molecular recognition capabilities due to their exceptional selectivity and sensitivity [14,15]. In MIP material systems, synthetic matrices resembling artificial antibodies have been engineered with precisely tailored recognition sites to detect specific target analytes [15,16]. Typically, the synthesis process involves strategically polymerizing carefully selected functional monomers and cross-linking agents around template molecules to create highly specific recognition cavities [15]. Following template extraction, the resulting rebinding sites highlight complementarity in terms of spatial architecture, molecular conformation, and functional groups with the target analytes, enabling highly specific molecular recognition events. Moreover, MIPs demonstrate remarkable chemical and thermal stability, exhibiting extended shelf-life and operational durability when incorporated through appropriate molecule immobilization and integration protocols [15]. Additionally, the extensive selection of monomers provides remarkable flexibility in the synthesis process, enabling the accommodation of diverse template molecules. The strengths of MIP technologies, combined with the advantages of electrochemical sensing methods, offer operational simplicity, rapid response times, and exceptional sensitivity. This synergy has the potential to drive the development of high-performance sensing platforms tailored to specific target applications. However, integrating MIPs with diverse biosensing modalities, such as electrochemical, optical, or electrical sensors, remains a challenge, particularly in preserving their molecular recognition capabilities [17]. Addressing these challenges involves advanced techniques, including compatible immobilization methods (e.g., surface grafting or functionalization), which ensure stable and efficient MIP integration [18,19]. Furthermore, the incorporation of hybrid materials, such as conductive polymers and nanocomposites, can effectively bridge the gap between the molecular recognition layer and the signal transduction elements, enhancing overall sensor performance [20,21]. Despite these advances, limited progress has been made in leveraging MIP-based technologies for detecting salivary biomarkers linked to specific diseases, highlighting a critical area for further research and innovation in biosensing platforms [22].

**Scheme 1:**
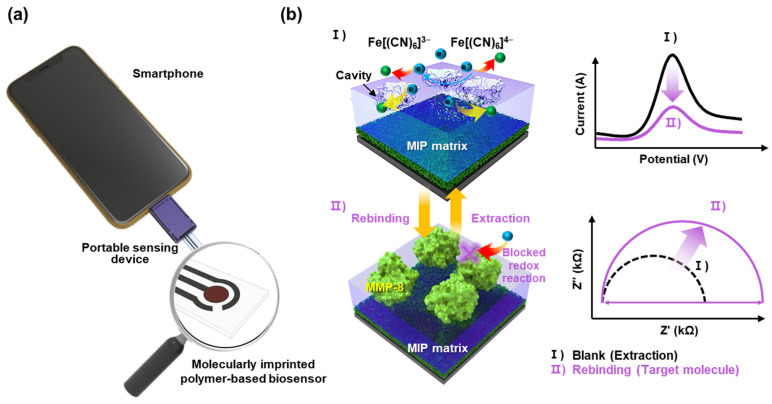
(**a**) Schematic illustration of a mobile POC device platform for periodontitis diagnostics. (**b**) MIP-based biosensor platform for the selective detection of the salivary biomarker MMP-8, highlighting the imprinting, extraction, and rebinding processes.

Herein, we present a strategic framework for the development of a highly responsive biosensor platform for periodontitis diagnostics, effectively mediating MIP/analyte interactions to enable the rational design of an active MMP-8 sensing system. In the proposed materials combination, MIPs are functionalized with conductive nanomaterials to enhance signal transduction efficiency. Our innovative approach improves the binding interaction between the MIP electrodes and sensing cavities based on the electrochemical detection mechanism, resulting in precise and measurable outputs. Since usually generated MIPs inherently lack signal-transducing properties, establishing an effective interface with the sensing element is a pivotal aspect in the design and optimization of such systems [21,23]. To further stabilize electrochemical performance and strengthen the formation of MIP architectures, partially reduced graphene oxide (rGO) was electrodeposited as a conductive support matrix onto carbon electrode surfaces prior to the fabrication of the MIP layer. For molecular design, eriochrome black T (EBT) was selected as a functional monomer due to its versatile chemical functionalities, including nitro, sulfonic acid, and hydroxyl groups, which facilitate the creation of high-affinity complementary binding sites through specific interactions with the target protein [23]. Additionally, molecular dynamics (MD) simulations were conducted to predict binding specificity, mapping the non-covalent bonding energies between the EBT monomer and the MMP-8 protein sequence. These simulations effectively guided the design of MIPs with enhanced binding affinities and selectivity for the target protein. Thus, the selective molecular recognition capabilities of the developed MIP-based biosensor for MMP-8 detection were comprehensively evaluated using electrochemical techniques, including electrochemical impedance spectroscopy (EIS) and square wave voltammetry (SWV). Furthermore, the proposed system can potentially be integrated with mobile devices [24], enabling the creation of a POC testing platform specifically tailored for the detection of the MMP-8 biomarker in periodontal disease diagnostics by robustly demonstrated detection mechanism, as illustrated in Scheme 1. By combining innovative MIP materials with advanced surface modification strategies on sensing electrodes, this work highlights the viable route of MIP-based biosensors to deliver developed sensing performance in analytical environments, paving the way for the practical application in clinical diagnostics.

## 2. Results and Discussion

### 2.1. Fabrication of EBT/GO-Based MIP Electrodes Using Electropolymerization

**Figure 1:**
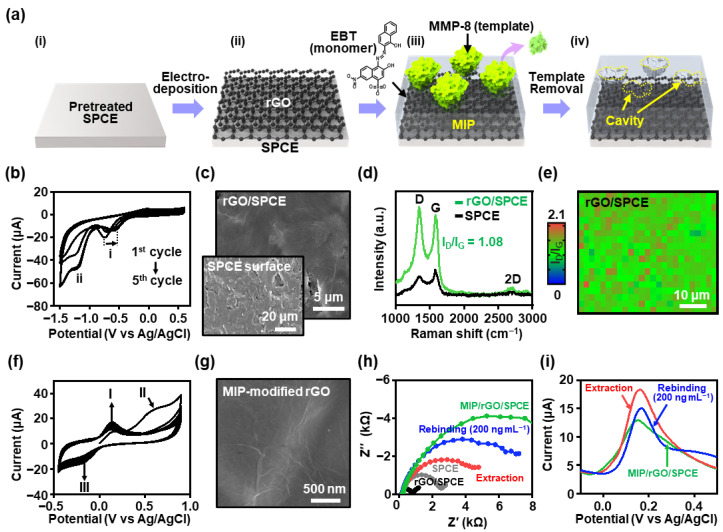
(**a**) Schematic illustration of the sequential process fabricating MIP-based electrode for MMP-8 biomarker detection. The overall process incorporates the following steps: (i) the pretreatment of SPCE, (ii) the electrodeposition of GO on the pretreated SPCE, (iii) imprinting process by electrodeposition of PEBT film in the presence of MMP-8 template, and (iv) removal of the MMP-8 template from the MIP matrix. (**b**) CV curves during the electrodeposition of GO on the pretreated SPCE in a 0.01 M PBS containing 0.1 M LiClO_4_. (**c**) SEM images of GO-modified SPCE as WE (inset image: the surface morphology of bare SPCE). (**d**) Raman spectra of the pretreated SPCE and the GO-modified SPCE measured in the range from 1000 to 3000 cm^−1^. (**e**) The Raman mapping of the rGO/SPCE surface. (**f**) CV curves during the electropolymerization of EBT functional monomer in the presence of the MMP-8 template on the rGO/SPCE in a 0.1 M PB solution containing 1% *v/v* ACN. (**g**) SEM image of the MIP/rGO/SPCE surface. EIS responses (**h**) and SWV measurements (**i**) for stepwise-modified MIP electrodes; all measurements were performed in KCl solution (0.1 M) containing 5 mM [Fe(CN)_6_]^3−^/^4−^.

Figure 1a illustrates the sequential process for fabricating an MIP-based biosensor designed for the detection of MMP-8. The synthesis of the MIP-based working electrode (WE) involves several key steps of surface pretreatment on the screen-printed carbon electrode (SPCE), electrochemical deposition of the GO interlayer, electropolymerization of the EBT-based MIP (i.e., PEBT), and template extraction from the MIP matrix. In the initial stage of MIP film formation, the SPCE surface was electrochemically activated in a 0.01 M PBS solution through successive CV cycles (Figure 1(ai)). This step is crucial to remove binders and additives in the carbon ink used in the screen-printing process, which can hinder electron transfer on the electrode, as an important factor for the functionality of electrochemical sensors [25,26]. Surface pretreatment plays a critical role in enhancing the electrochemical properties of the WE by improving electron transfer kinetics, lowering charge transfer resistance, increasing surface conductivity, and promoting more efficient redox probe reactions. Accordingly, precise control and optimization of the pretreatment process are essential to ensure consistent sensor performance. Following electrochemical activation, the WE surface was modified via CV cycling in a GO solution, as illustrated in Figure 1(aii). GO, characterized by its 2D carbon lattice composed of conjugated hexagonal rings and oxygen-containing functional groups, such as hydroxyl, epoxy, carboxyl, and carbonyl moieties, facilitates intermolecular interactions through hydrogen bonding and π-π stacking [27]. Upon electrochemical deposition, the GO layer effectively modified the SPCE surface with a smooth interface, providing an impedance-tunable interlayer for stable MIP formation. In other words, this GO layer facilitates the development of additional uniform deposition by interacting with the EBT monomer, containing the MMP-8 template protein [28,29].

In our material system, to construct the MIP layer, EBT was selected as the functional monomer due to its diverse reactive groups, which enable specific non-covalent interactions with the MMP-8 target protein. The EBT-based MIP films were electrochemically polymerized onto the GO-modified SPCE surface in a 0.1 M phosphate buffer (PB) solution, containing 0.58 µM MMP-8 and 1.0 mM EBT. Polymerization of EBT was conducted under a specific CV cycle at a scan rate of 100 mV s^−1^ within a voltage range of −0.45 V to +0.90 V over 8 cycles (Figure 1(aiii)). The final step involved the extraction of the MMP-8 template to form imprinted cavities in the MIP matrix. An acetonitrile (ACN) solution (0.2 M) was applied to the MIP electrode surface, followed by 25 CV cycles in the voltage range of −0.8 V to +0.2 V at a scan rate of 100 mV s^−1^ (Figure 1(aiv)). At this template extraction step, the mixed solvent of ACN and water induced polymer film swelling, increased polymer chain spacing, and weakened interactions between the MMP-8 template and PolyEBT (i.e., PEBT), allowing efficient template removal and imprint cavity formation [30,31]. Therefore, during this process, noncovalent bonds between the PEBT matrix and the MMP-8 protein were cleaved by repulsive force, thereby completely removing them and leaving behind sterically and chemically complementary imprint cavities with target protein. Moreover, to evaluate and compare MIP-based sensor performance, non-imprinted polymer (NIP) samples were also prepared using identical procedures without the MMP-8 template. As noted, the optimized MIP fabrication process yielded a ‘molecular memory’ sensor with surface-imprinted PEBT films, capable of selectively capturing target molecules with high affinity when exposed to aqueous analytes containing MMP-8 protein.

### 2.2. Surface and Electrochemical Characteristics of rGO/SPCE and MIP/rGO/SPCE

In this study, the electrochemically deposited GO in a partially reduced form (i.e., rGO) played an important role in the MIP-based sensing mechanism, distinguishing it from other previously explored electrochemically deposited molecular imprinting techniques. To elucidate the significance of each step, sequential surface analyses for rGO deposition and MIP polymerization were performed separately (Figure 1b–g). Figure 1b illustrates a cyclic voltammogram depicting the electrochemical reduction of GO. To support the reduction of GO, 0.1 M of LiClO_4_ was dissolved in a 0.01 M PBS to facilitate redox mediation and pH buffering effect at the same time, allowing the reaction progress to be clearly observed in each cycle. One anodic peak and two cathodic peaks (i and ii) were observed, where the continuous increase in cathodic current at peaks corresponds to the gradual reduction of GO, signifying the progressive accumulation of conductive rGO. The peaks from (i) and (ii) primarily represent the reduction of oxygen-containing functional groups such as phenolic hydroxyl, carboxyl and epoxy groups [32,33,34]. Specifically, the reduction of the C–O group occurs at approximately −0.75 V in the first cycle; in subsequent cycles, however, the potential shifts towards the anode. This suggests that the amount of chemical energy required to reduce the C–O group decreases as GO stacks. Similarly, the C=O group reduction, which is typically reduced at approximately −1.2 V, was not observed in the first cycle due to a relatively high chemical energy barrier, but appeared from the second cycle onwards. Afterwards, the GO layer had a significant influence on the further reduction process. As a result, electrical current gradually increased during the deposition cycle, forming a compact film structure [34]. This experimental result can be a useful guideline for preparing a GO-modified electrode that has a beneficial influence on the electrode’s structural configuration and electrochemical properties, providing an abundant active site for the EBT polymerization.

The SEM images in Figure 1c further reveal the successful deposition of rGO film, representing characteristic nanowrinkles and crumpled structures on the surface morphology of rGO-modified SPCE, as typically observed in 2D film materials [35]. Compared to the pristine SPCE surface, the deposited rGO film exhibited a smooth structure with the stacked rGO sheets, which can be advantageous for the electrochemical deposition of PEBT [36]. Importantly, the activated SPCE surface with a pretreatment ensured uniform GO deposition, facilitating the continuous formation of a rGO film despite the inherent irregular nanostructures [36]. Raman spectroscopy was used to explore structural changes in the deposited rGO layer (Figure 1d), where key peaks corresponding to the D band (1340 cm^−1^) and G band (1582 cm^−1^) were observed, representing structural defects and sp^2^-bonded carbon vibrations [37,38,39]. The increased intensity ratio of the D and G bands (I_D_/I_G_) from ~0.66 to ~1.08 confirmed the defect generation during the GO reduction process [39]. Raman mapping image in Figure 1e also demonstrated relatively uniform distribution of the D and G band intensities across the rGO/SPCE surface, indicating a laterally homogeneous WE film. In the initial experimental design, we assumed that this surface modification based on rGO would be a critical factor in ensuring the consistency of electrochemical properties and sensor responses. In addition, XPS further supports the expected surface chemistry, as summarized in Figure S1. The C 1s envelope (Figure S1a) is deconvoluted into sp^2^ C–C at ~284.8 eV (asymmetric line shape), C–O/C–OH at ~286.2–286.8 eV, C=O at ~287.0 eV, and O–C=O at ~289.0–289.5 eV, together with a π→π* shake-up feature at ~291–292 eV characteristic of conjugated sp^2^ domains [40,41]. In the O 1s region (Figure S1b), components assigned to carbonyl/quinone O (C=O, ~531.0–531.8 eV), C–O(H)/epoxy (~532.3–533.0 eV), and a high-BE shoulder from adsorbed/intercalated H_2_O (~533.5–534.0 eV) indicate that oxygenated groups remain, consistent with partially reduced GO [34,41]. The N 1s spectrum was deconvoluted into two dominant components at 399.6 eV (pyrrolic/amide-type N–(C)_2_) and 400.9 eV (graphitic N), along with a minor peak at 399.2 eV attributed to C=N–C and a π–π shake-up satellite (Figure S1c). These results indicate that the rGO substrate is partially nitrogen-doped, which may enhance charge transfer and introduce redox-active defect sites [42,43,44]. Electrochemical data corroborate these assignments. In 0.1 M KCl with 5 mM [Fe(CN)_6_]^3−/4−^, square-wave voltammetry (SWV) exhibits a ~50 mV cathodic shift in the oxidation peak and a ~2.2-fold increase in peak current after GO deposition, followed by electrochemical reduction to rGO (Figure S2b). Complementarily, EIS shows a near-vanishing charge-transfer resistance upon rGO modification (Figure S2a). These results indicate that the porous, conjugated rGO film enlarges the electroactive area and accelerates heterogeneous electron transfer, providing a robust and reproducible baseline for subsequent MIP construction [45].

Based on the collective data, we propose the following working hypothesis: the presence of basic and conjugated nitrogen species in the rGO substrate (i) enhances the intrinsic conductivity of the rGO layer and (ii) lowers the activation energy for azo-bond oxidation in EBT, thereby modulating the electropolymerization mechanism responsible for forming the molecularly imprinted film. MIP/rGO/SPCE electrodes were fabricated via CV in a mixed solution containing MMP-8 template proteins and EBT monomers, as schematically illustrated in Figure 1a and electrochemically characterized in Figure 1f. During the electropolymerization process, two prominent redox peaks denoted as Peak I and Peak III were observed at +0.15 V and −0.37 V, respectively. These peaks correspond to reversible phenoxyl-radical coupling reactions between the benzoquinone diimine structures and EBT monomers, consistent with prior studies [46]. Additionally, an anodic peak (Peak II) appeared at +0.58 V, which is plausibly attributed to the one-electron oxidation of pre-oxidized azo groups, potentially followed by partial N–N bond cleavage to generate reactive radical intermediates [47]. These radicals are likely stabilized or guided by the electronic environment of the N-doped rGO surface [48], facilitating covalent bonding with carbon atoms adjacent to pyridine- or pyrrole-type nitrogen sites [49]. This interaction supports an alternative grafting pathway for EBT, deviating from traditional electropolymerization mechanisms [30]. Notably, the intensity of Peak II diminished rapidly with increasing cycle number, suggesting progressive film growth and steric hindrance, while also indicating that the rGO underlayer effectively modulates polymer film formation. In contrast, the peak currents associated with Peaks I and III gradually increased with successive CV cycles, implying continued monomer incorporation and film thickening. This monotonic increase, despite the film growth, highlights the low charge-transfer resistance maintained by the conjugated nature of the PEBT structure and the conductive rGO interlayer, resulting in an expanded effective electroactive surface area [31,50]. These distinct polymerization behaviors, divergent from previous electropolymerization profiles on non-modified substrates, clarify the role of the rGO layer not merely as a conductive support but as an active modulator of polymerization kinetics and imprinting site fidelity. This effect contributes to enhanced sensor performance for MMP-8 detection. Supporting this hypothesis, the SEM image in Figure 1g reveals that the PEBT-based MIP films formed on rGO/SPCE possess a wrinkled morphology closely following the underlying rGO surface. Comparable morphologies were observed in NIP films (Figure S3), confirming uniform film deposition. Consequently, these morphological and electrochemical characterization analyses validate the strategic use of rGO as an interfacial layer to direct MIP electropolymerization pathways for enhancing molecular recognition capabilities.

### 2.3. Electrochemical Properties of PEBT/rGO/SPCE-Based MIP Electrodes

To investigate the stepwise electrochemical behavior and interfacial characteristics of each functional layer deposited on the SPCE surface, EIS and SWV were performed in 0.1 M KCl solution containing 5 mM [Fe(CN)_6_]^3−^/^4−^. EIS measurements were conducted at 0.1 V with a 10 mV amplitude across a frequency range of 0.1 Hz–100 kHz. The corresponding Nyquist plots are shown in Figure 1h. The imaginary impedance (−Z″) peak from the Nyquist plot served as a key metric for evaluating interfacial charge-transfer resistance [51]. For the pretreated SPCE, the −Z″ peak was ~1020 Ω (gray line), which markedly decreased to ~445 Ω following rGO deposition (black line), indicating enhanced electron transfer due to the high conductivity and interface-modifying characteristics of rGO. Upon electropolymerization of the MIP film in the presence of MMP-8 templates, the −Z″ value increased substantially to ~4110 Ω (green line), reflecting the insulating nature of the PEBT matrix, which hinders diffusion of redox probes [30,31]. Subsequent removal of the MMP-8 templates via ACN washing reduced the impedance to ~1810 Ω (red line), attributed to the formation of recognition cavities that facilitate ion diffusion. To assess the performance of the MIP electrode, a representative concentration of 200 ng mL^−1^ MMP-8 protein in 0.5 M Tris-HCl buffer was introduced to induce rebinding on the recognition sites formed on the MIP film surface. This rebinding process spontaneously blocked the migration of probe ions, causing the impedance peak value to increase to ~2890 Ω (blue line). As a control, a NIP electrode fabricated under identical conditions but without the MMP-8 template exhibited negligible impedance changes upon analyte exposure (Figure S4), confirming the absence of specific binding sites. The slight impedance reduction observed during the ACN treatment step in the NIP was likely due to minor surface reorganization and was not indicative of template-related binding [30,31]. Complementary SWV measurements (−0.2 V to +0.5 V), shown in Figure 1i and Figure S5, further validated these findings. The initial MIP-modified rGO electrode exhibited suppressed peak current due to the nonconductive nature of the polymer. After template removal, an increase in current was observed, consistent with the creation of recognition cavities. Rebinding of MMP-8 led to a decreased current, supporting selective occupancy of the imprinted sites. In contrast, the SWV response of the NIP electrode remained largely unchanged throughout the entire sequence, reflecting the absence of specific recognition interactions. Thus far, as summarized in Figure 1h,i, the preliminary measured electrochemical behaviors of the MIP electrode confirmed the successful construction of the molecular sensing electrode that incorporates specific recognition sites (i.e., selective detection of MMP-8 protein), demonstrating its effectiveness and reliability for biosensing applications. However, electrochemical sensing of protein-polymer interactions may be susceptible to false-positive signals under suboptimal pH or ionic strength conditions [52,53,54,55]. To address this, we evaluated the MIP/NIP response in three electrolyte buffers (i.e., 0.1 M KCl (pH 6.0), 0.01 M PBS (pH 7.4), and 0.5 M Tris-HCl (pH 8.0)) chosen to span the vicinity of the isoelectric point of MMP-8 (~6.4) [31]. Normalized response values of MIP versus NIP are shown in Figure S6. Among these, Tris–HCl provided stable and conservative binding signals, while KCl offered higher electrochemical sensitivity. Full discrimination between binding and transduction mechanisms would benefit from additional controls, including ionic-strength variation, alternate redox probes, or probe-free impedance modalities.

### 2.4. Surface Characterization of MIP and Extracted MIP Electrodes

**Figure 2:**
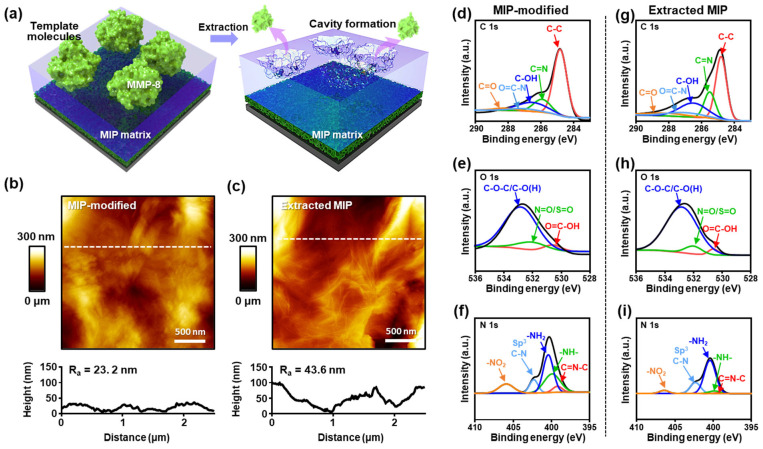
Structural and chemical validation of molecularly imprinted cavity formation on PEBT/rGO/SPCE. (**a**) Schematic representation of the MMP-8–MIP interface before and after template extraction, illustrating the formation of recognition cavities following MMP-8 removal. AFM topographies of the MIP film before (**b**) and after (**c**) template extraction, showing a notable increase in surface roughness from RMS = 23.2 nm to RMS = 43.6 nm. Dashed lines indicate the cross-sections used for height profile analysis (lower panels). (**d**–**f**) High-resolution XPS spectra of C 1s, O 1s, and N 1s regions for the MIP-modified electrode, prior to template removal. (**g**–**i**) Corresponding spectra after extraction reveal pronounced changes in the nitrogen chemical environment, consistent with the removal of protein-derived functional groups.

To systematically evaluate the imprinting outcome and complement the electrochemical results discussed in Section 2.3, we analyzed the PEBT-based MIP surface before and after template extraction, an essential step in MIP-based sensor fabrication, highlighted in Figure 2a. Atomic force microscopy (AFM) and high-resolution XPS were employed to indirectly confirm the successful formation of recognition cavities following template removal. AFM provided quantitative surface topography and roughness measurements, while XPS was used to track changes in chemical composition associated with protein removal. Thus, these surface analyses offer complementary insight into cavity formation and molecular specificity enabled by the imprinting process [56]. AFM images collected before and after MMP-8 template extraction (Figure 2b,c) revealed distinct morphological changes. The root-mean-square (RMS) surface roughness of the MIP film prior to extraction was ~23 nm, which increased to ~43 nm after extraction. This increase in surface roughness is indicative of cavity formation at the PEBT film surface, consistent with the removal of MMP-8 templates. These results align with previous studies on protein imprinting, where roughness increases serve as a marker of successful template removal and cavity formation [31,57]. Notably, the contrast between MIP and NIP film surfaces reflects the fine control achieved during imprinting and highlights the functional specificity introduced by protein templating. To investigate the chemical evolution of the MIP surface during imprinting and extraction, high-resolution XPS was performed at each fabrication stage. Although small protein biomolecules present challenges in directly measuring structural changes or detecting residual protein forms within the MIP matrix after extraction, surface-sensitive techniques such as XPS allow tracking of indirect markers [17,58]. The C 1s spectra (Figure 2d,g) showed characteristic peaks corresponding to C–C (~284.8 eV), C–OH (~286.5 eV), O=C–N (~287.0 eV), and C=O (~288.2 eV). Likewise, the O 1s spectra (Figure 2e,h) featured peaks attributed to C–O–C/C–OH (~532.8 eV), N=O/S=O (~532.0 eV), and O=C–OH (~530.6 eV). Although surface chemistry changed substantially after the PEBT polymerization, direct assessment of template removal based on these spectra was limited due to overlapping features and broad peak envelopes.

To resolve the limitations of C 1s and O 1s spectra in distinguishing protein extraction, the N 1s region was identified as a more sensitive and specific marker for monitoring molecular imprinting fidelity (Figure 2f,i). Deconvolution of the high-resolution N 1s spectra revealed five major components: C=N–C (~399.2 eV), –NH– (~399.9 eV), –NH_2_ (~400.4 eV), sp^3^ C–N (~402.2 eV), and –NO_2_ (~405–407 eV). Among these, the –NH– peak significantly decreased after template extraction, strongly indicating the removal of proteinaceous nitrogen functionalities and thus verifying successful imprint cavity formation [58]. Concurrently, a slight decrease in –NO_2_ intensity and a corresponding increase in the fractional –NH_2_ contribution were observed. This redistribution is attributed to a combination of template removal and partial electrochemical or solvent-driven transformation of azo/nitro groups during the extraction cycles [46]. Additionally, the C=N–C peak, characteristic of azo–diimine linkages, diminished nearly to baseline levels post-extraction, supporting the hypothesis that these reactive sites either participate in radical grafting during polymerization or undergo reductive cleavage upon exposure to ACN or applied potential. This loss likely contributes to the apparent increase in free –NH_2_ content. In contrast, the sp^3^ C–N signal (~402.2 eV) remained essentially unchanged, indicating structural stability of the underlying nitrogen-doped rGO/PEBT matrix, as corroborated in Section 2.2. These component-level shifts were substantiated by a ~52.2% decrease in the total N 1s atomic percentage after extraction (Table S1), offering robust quantitative evidence of successful template removal and cavity generation within the MIP matrix [30,58]. In contrast, the non-imprinted control (NIP/rGO/SPCE) exhibited a distinctly different chemical profile (Figure S7). The broad C–OH peak in the C 1s region diminished, while the relative intensities of O=C–N and C=N increased. Notably, the N 1s spectrum of the NIP electrode retained a higher –NH_2_ contribution and showed minimal –NH– content, supporting the interpretation that the –NH– signal in MIP arises primarily from protein-derived moieties. The NIP also exhibited a prominent –NO_2_ signal and enhanced O=C–N binding in C 1s, consistent with a more oxidized, densely crosslinked network formed via untemplated EBT polymerization. This denser, imine/nitro-rich matrix likely possesses fewer polar donor–acceptor sites and limited H-bonding capacity. Taken together, these results demonstrate that the MIP matrix, prior to extraction, contains a broader spectrum of H-bonding groups (notably broader and stronger C–OH) and protein-specific nitrogen features (–NH–), which are selectively removed through template extraction to yield well-defined recognition cavities. The surface chemical changes observed across the C, O, and especially N 1s regions directly support the formation of functional, protein-selective binding sites. These findings clarify the electrochemical data, offering a stepwise molecular-level confirmation of the imprinting mechanism and the functionality of the PEBT/rGO/SPCE-based biosensor platform.

### 2.5. Computational Characterization of Binding Specificity Between EBT and MMP-8

**Figure 3:**
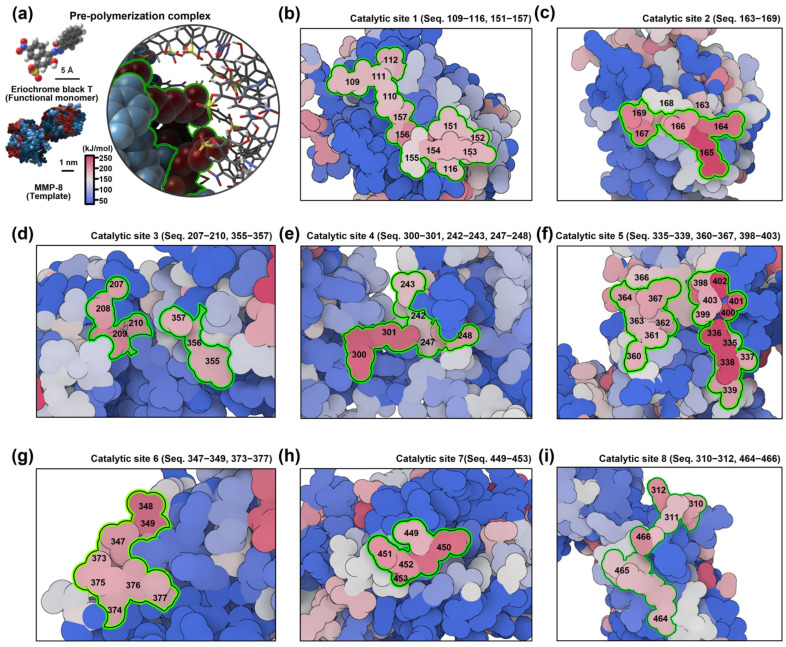
Sequence- and electrostatic potential-resolved identification of MMP-8 surface regions contributing to molecular imprinting. (**a**) Molecular ESP mapping of MMP-8 rendered on its solvent-accessible surface. The red-to-blue color scale indicates negative to positive ESP, respectively. The green outline highlights the pre-polymerization recognition region targeted by EBT monomers, centered on catalytic site 8 (C8) within the hemopexin-like domain. The inset shows the representative DFT-modeled pre-polymerization complex, in which the EBT molecule is oriented toward catalytically active residues within C8, suggesting favorable binding energetics during imprint formation. (**b**–**e**) Catalytic domain-derived ESP-defined recognition sites: (**b**) C1 includes residues 109–116 and 151–157; (**c**) C2 spans residues 163–169; (**d**) C3 comprises residues 207–210 and 355–357; (**e**) C4 includes residues 242–248 and 300–301. (**f**–**i**) Hemopexin domain–associated β-propeller loop regions showing high ESP and accessibility: (**f**) C5 (residues 335–339, 360−367, 398–403); (**g**) C6 (residues 347–349 and 373–377); (**h**) C7 (residues 449–453); and (**i**) C8 (residues 310–312 and 464–466), which showed the strongest local binding energy and spatial compatibility with the EBT monomer.

Building upon the experimental validation of protein imprinting presented in the previous section, we conducted a computational analysis to assess the molecular-level binding specificity between EBT and MMP-8. In molecular imprinting, selective rebinding is primarily governed by the geometric complementarity and binding energetics of the pre-polymerization complexes [31]. The recognition sites that emerge post-polymerization are essentially immobilized replicas of these transient, noncovalent interactions [59]. To examine this specificity, we employed density functional theory (DFT) simulations to systematically map the interaction landscape between EBT and MMP-8 (Figure 3a) [60,61]. As illustrated in Figure S8a, the structural model of MMP-8 was derived from the AlphaFold-based predictions (UniProt ID: P22894) to refine flexible and unresolved regions [61,62,63,64,65]. MMP-8 consists of three distinct domains: a propeptide domain (residues 1–108), a catalytic domain (residues 109–275), and a hemopexin-like domain (residues 276–467) [66,67]. The propeptide domain, which contains linear peptide motifs responsible for N-terminal capping and peptidoglycan interactions, is cleaved during activation. The catalytic domain harbors the active site containing a zinc ion coordinated by a canonical HEXXHXXGXXH motif, essential for proteolytic activity. The hemopexin-like domain adopts a four-bladed β-propeller structure and is conserved among MMPs, functioning in substrate recognition and inhibitor binding. Given that the catalytically active form of MMP-8 is implicated in inflammatory diseases such as periodontitis, our simulations focused on this activated configuration.

To quantitatively evaluate EBT-MMP-8 interactions, we calculated the noncovalent binding energies between EBT and all twenty amino acids individually, as found throughout the full-length MMP-8 sequence (467 residues). The binding energy of each pre-polymerization complex was computed using the polarizable continuum model and defined as

Binding energy (Δ*E*) = *E*_Complex_ − *E*_EBT_ − *E*_Amino acid_

where each *E* represents the electrostatic potential (ESP) energy mapped on the van der Waals surface of the molecule [60,61]. These values are visualized in Figure S8b, which is based on the hydrogen-bonding interactions between the amino acids and the EBT (see Table S2). By excluding non-neighboring long-range interactions, our analysis focused on the most relevant spatially localized binding interactions that contribute to site formation [59,60,61,62]. The spatial arrangement of EBT monomers within the pre-polymerization ensemble reveals distinct energetic preferences across the MMP-8 surface. This arrangement enables discrimination between catalytically relevant regions and off-target sites, while preserving the protein’s conformational integrity during polymerization. As shown in Figure 3b–i, ESP surface mapping identified eight discrete regions exhibiting high-affinity EBT binding potential (Supplementary Video S1). These regions were distributed across both the catalytic and hemopexin-like domains. Notably, four of these regions (denoted as C1–C4) are located within or adjacent to the catalytic cleft, where they are well-aligned with the zinc active site and the substrate-binding S1′ subsite [63,64]. This alignment includes key residues such as Arg222 and Tyr227, which are known to influence ligand selectivity in MMPs. The observed polarity and curvature of the ESP in the C1–C4 regions indicate favorable local electrostatic and hydrogen-bonding interactions with functional groups of the EBT monomer, including hydroxyl, azo (–N=N–), nitro (–NO_2_), and sulfonate (–SO_3_^−^) moieties [64,65,66]. These interactions provide a plausible mechanistic basis for the formation of multi-point pre-polymerization complexes within the catalytic groove. During electropolymerization, these complexes are immobilized within the polymer matrix, ultimately leading to the formation of selective, high-affinity recognition cavities after template extraction [61,66,67]. In contrast, the remaining four patches (C5–C8, Figure 3f–i), located on the hemopexin-like domain, exhibited more dispersed ESP profiles and were situated along the β-propeller interfaces. Among these, the C8 region was found to have one of the strongest local binding energies. However, rather than serving as a primary recognition site, this region likely functions as an anchoring or orientation motif that guides EBT alignment and facilitates initial complexation. This interpretation is consistent with the established domain-specific functions in MMPs, wherein the hemopexin-like domain contributes to substrate positioning and interdomain coordination, while the catalytic cleft governs fine chemical selectivity [67,68]. Taken together, these computational results suggest that the catalytic sites C1–C4 are the primary contributors to high-affinity, selective cavity formation, while sites C5–C8 serve auxiliary roles in stabilizing or orienting the pre-polymerization complex. These findings suggest the energetic heterogeneity across the MMP-8 surface and highlight how distinct pre-polymerization arrangements may influence the fidelity, reproducibility, and sensing performance of the resulting MIP system. Furthermore, the detailed ESP mapping and binding energy quantification presented here offer valuable design criteria for optimizing monomer–template interactions in MIP-based biosensor platforms [17,31,69].

### 2.6. Optimization of Fabrication Parameters for Enhanced Sensing

**Figure 4:**
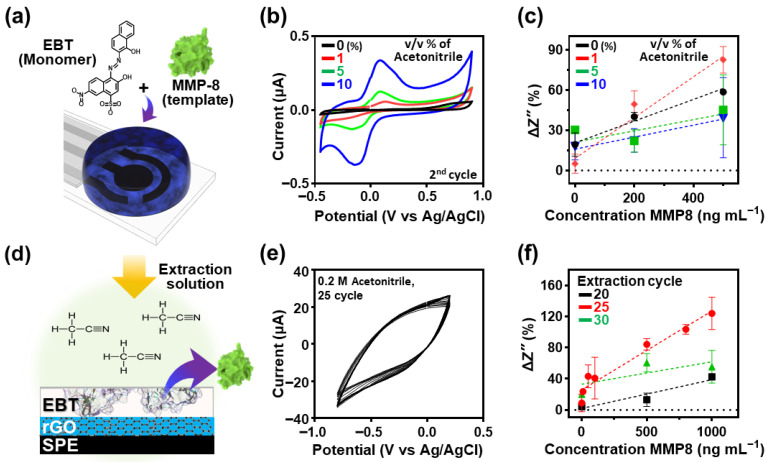
Optimization of fabrication parameters for improved sensor performance. (**a**) Schematic representation of the electropolymerization process on an rGO/SPCE, where EBT monomers form a MIP matrix around the MMP-8 template. (**b**) CV profiles showing the effect of different ACN volume fractions (1%, 5%, and 10% *v*/*v*) in the pre-polymerization mixture on the electropolymerization behavior. (**c**) MMP-8 concentration-dependent sensor responses of PEBT/rGO/SPCE fabricated under different ACN conditions, highlighting optimal performance at 1% ACN due to balanced monomer solubility and cavity formation. (**d**) Schematic depiction of the template extraction step performed using 0.2 M ACN as an elution solvent via electrochemical cycling. (**e**) CV profiles during template removal, demonstrating progressive extraction efficiency with increasing scan cycles (20, 25, 30 cycles). (**f**) Sensory response of MIP sensor (ΔZ″ vs. concentration) to various concentrations of target molecule (0–1000 ng mL^−1^) following different extraction cycles, indicating that 25-cycle extraction yielded the highest signal response, while 30-cycle extraction showed degradation, suggesting overextraction compromises MIP integrity.

Figure 4a schematically depicts the fabrication process of the PEBT/rGO/SPCE-based MIP biosensor using a three-electrode electrochemical setup comprising a WE, counter electrode (CE), and reference electrode (RE). The synthesis of the MIP sensing layer was initiated by applying a droplet of a pre-polymerization mixture onto the electrodes’ surface, following the computationally identified monomer–template interactions described in Figure 3. In this section, we focus on the systematic optimization of fabrication parameters aimed at enhancing the fidelity and sensitivity of molecular recognition sites for the selective detection of MMP-8. A key variable investigated was the solvent composition, specifically the volume fraction of ACN, which directly influences monomer solubility and film formation. To evaluate this, the polymerization process was carried out using varying ACN concentrations (i.e., 1%, 5%, and 10% *v*/*v*) as the solvent for EBT, and the resulting electrochemical responses were monitored via CV (Figure 4b). ACN was selected as the solvent due to its high polarity and aprotic nature, which facilitates the solubilization of EBT and promotes homogeneous distribution of functional monomers during polymer film growth [70]. This parameter is particularly critical for ensuring uniform pre-polymer complex formation and controlling the microstructure of the imprinted cavities post-polymerization. The MIP electrodes were generated on rGO-modified SPCE through electrochemical polymerization using a finely tuned solution, containing 0.1 M phosphate buffer, 0.58 × 10^−6^ M MMP-8, and 1.0 × 10^−3^ M EBT, and the CV parameters included a potential range of −0.45 to +0.90 V, and a scan rate of 100 mV s^−1^ (8 cycles).

Figure 4c illustrates the sensory response characteristics of the MIP electrodes to varying MMP-8 concentrations (0, 200, and 500 ng mL^−1^) as a function of the ACN volume fraction in the polymerization solution. While the PEBT-based MIP electrodes prepared without ACN exhibited moderate linear sensitivity, optimal sensing performance was achieved with 1% ACN, which enhanced recognition site formation. In contrast, higher ACN concentrations (5% and 10% *v*/*v*) disrupted the polymerization rate, interfering with proper cavity formation, as evidenced by the diminished recognition sensitivity compared to the optimized condition. These results showed a critical role of ACN content in modulating MIP film structure, particularly through its effect on monomer solubility and polymerization dynamics. To further investigate sensor performance, we evaluated the impact of template removal conditions on sensing behavior. As presented in Figure 4d,e, template extraction was carried out via CV in a 0.2 M ACN solution under slightly modified parameters, including a potential window of −0.8 to +0.2 V and a scan rate of 100 mV s^−1^, based on previously reported protocols [30]. Figure 4f summarizes the concentration-dependent sensor response (ΔZ″ vs. concentration) following different extraction cycles. Up to 25 cycles, sensor performance was improved, with enhanced sensitivity, observed even at higher MMP-8 concentration range (i.e., 500 and 1000 ng mL^−1^). However, further extension to 30 cycles resulted in a decline in detection capability at the higher concentration range, suggesting degradation of the PEBT-based imprinting matrix. These results experimentally revealed that excessive cyclic extraction can damage the MIP structure, while insufficient extraction leaves residual template within the film, both of which impair sensor efficiency. Thus, the extraction process must be precisely controlled to preserve the integrity of the imprinted cavities and ensure high binding affinity. In addition, the temporal response characteristics of the optimized sensor were examined at a fixed MMP-8 concentration of 200 ng mL^−1^, as shown in Figure S9. Signal saturation occurred after approximately 50 min, indicating the minimum required time for effective analyte rebinding to the recognition sites. After optimization of extraction and rebinding process, MIP electrode exhibited an apparent two-regime response that is consistent with heterogeneous binding sites commonly observed in protein-imprinted electrodes. At low MMP-8 levels, high-affinity imprinted cavities dominate the uptake, showing a steeper initial slope; after partial saturation, signal changes are governed by lower-affinity and nonspecific sites, yielding a more gradual slope at higher concentrations [71]. Collectively, these results emphasize the importance of optimizing both the polymerization and template extraction conditions to achieve reproducible and stable sensing performance. Moreover, we established a practical benchmark for the minimum measurement time needed to ensure accurate MMP-8 detection with minimal variability, which can severely influence the characterization of statistical interpretation.

### 2.7. Electrochemical Sensing Performance Based on SWV Analytical Characteristics

**Figure 5:**
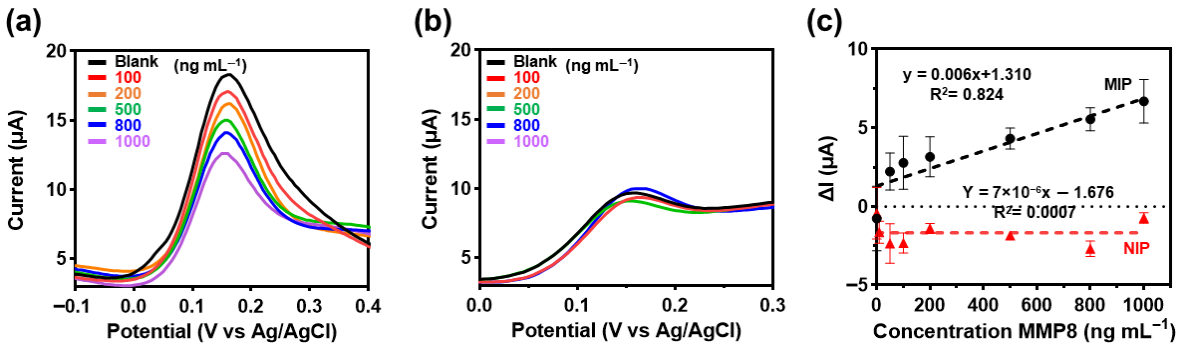
Electrochemical detection of MMP-8 using MIP-modified sensors. (**a**,**b**) SWV responses of MIP and NIP electrodes in 0.1 M KCl containing 5 mM [Fe(CN)_6_]^3−^/^4−^ upon incubation with increasing concentrations of MMP-8 (100–1000 ng mL^−1^); the MIP sensor exhibited concentration-dependent peak current variations, whereas the NIP showed minimal changes. (**c**) Normalized current changes (ΔI) as a function of MMP-8 concentration, confirming the enhanced sensitivity and binding specificity of the MIP sensor compared to the non-imprinted control.

To evaluate the optimized MIP-based biosensor, SWV method was employed, enabling rapid acquisition and analysis of data across a broad concentration range within a relatively short timeframe. This technique confirmed the sensor’s high sensitivity and linear response characteristics in concentration-dependent measurements, as illustrated in Figure 5. The detection experiment involved monitoring the current response to various concentrations (0, 100, 200, 500, 800 and 1000 ng mL^−1^) of MMP-8 solutions dispersed in a KCl electrolyte buffer solution (5 mM [Fe(CN)_6_]^3−^/^4−^) upon exposure to the MIP electrode surface. Based on the preliminary test conditions for rebinding and electrochemical characterization (see Figure S6), optimized parameters such as pulse amplitude, frequency and step potential were employed to ensure accurate analysis and eliminate environmental factors that could compromise the sensor’s analytical performance. Frequency primarily controls the balance between faradaic kinetics and capacitive charging (set by the RC time, film thickness, and redox diffusional length) within each half-period. At higher frequency, incomplete faradaic relaxation and larger capacitive contributions reduce peak height; at lower frequency, peaks broaden without a gain in sensitivity [70]. Furthermore, we optimized pulse amplitude to avoid the peak broadening (lower amplitude) and shift (higher amplitude) in the imprinted films [72]. A step height of 10 mV provided tens of data points across the oxidation peak, enabling robust peak picking. With our optimized measurement parameters, potential window −0.20 to +0.50 V (Ag/AgCl) fully spans the formal potential of [Fe(CN)_6_]^3−/4−^ on carbon (≈ +0.18–0.23 V) in this study.

As presented in Figure 5a, the SWV results revealed a gradual decrease in the current peak as MMP-8 concentration increased. This phenomenon is attributed to the specific binding of MMP-8 molecules within the molecularly imprinted cavities formed on the PEBT matrix on the electrode surface, which obstructed electron transfer between the redox probe in the electrolyte and the electrode surface. Thus, the efficient recognition and successive rebinding of MMP-8 were evidenced by the proportional relationship between the current suppression and analyte concentration. On the other hand, the NIP electrode displayed negligible current changes across the same analyte concentration range, as shown in Figure 5b. This outcome highlights the lack of specific binding sites on the NIP electrode, with only minor current variations caused by nonspecific physical adsorption.

The comparative analysis of MIP and NIP electrodes, conducted in the presence of redox probe ions, provided ΔI values as plotted in Figure 5c. The calibration curve for the MIP-based biosensor exhibited a strong linear relationship within the analyte concentration range of 0–1000 ng mL^−1^, with a correlation coefficient of R^2^ = 0.824, well-matched with primary objective of this study, which is to demonstrate quantitative performance within the abnormal clinical range (≥200 ng mL^−1^) of MMP-8 [73,74]. As presented, although the error bars in current data indicated slight variations, the trend of decreasing current with increasing concentration was clear and statistically significant. In contrast, the NIP electrode produced irregular current fluctuations and a significantly lower correlation coefficient (R^2^ = 0.0007), further confirming its inefficiency in specific binding. Additionally, the instability observed in the NIP electrode’s response, including minor desorption of MMP-8 molecules and fluctuating SWV currents, highlighted the superior susceptibility of the MIP electrode. The collective results imply the potential for the utilization of EBT through our newly designed polymerization pathway as a functional candidate for protein imprinting. Therefore, the prepared MIP electrodes demonstrated reproducible current responses that were directly proportional to the analyte concentration, validating their ability for accurate quantitative analysis within the broad concentration range of 0–1000 ng mL^−1^.

### 2.8. Impedimetric Analysis of MIP-Based Biosensor for MMP-8 Determination

**Figure 6:**
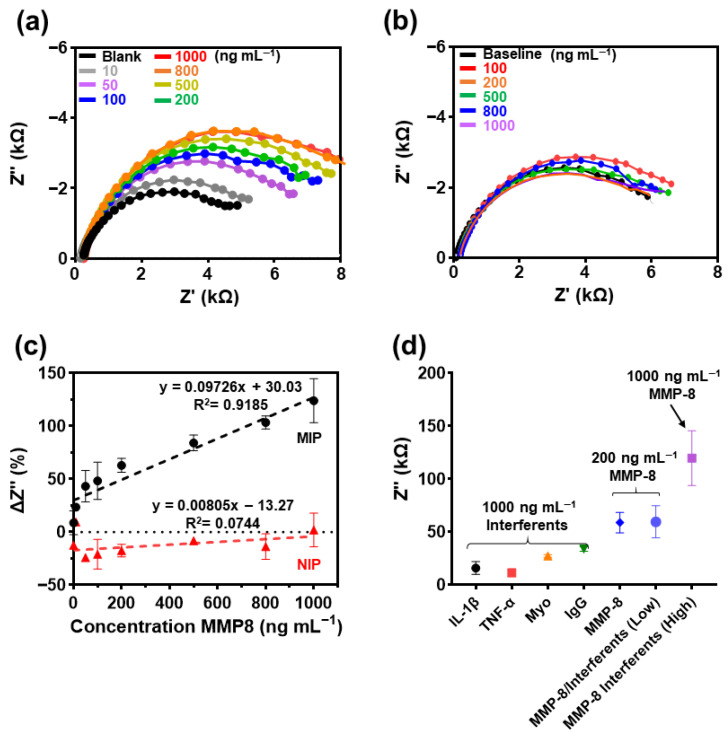
EIS analysis of MMP-8 detection performance and selectivity of the MIP-based biosensor. (**a**,**b**) Nyquist plots showing the impedance spectra of the fabricated MIP (**a**) and NIP (**b**) biosensors in response to increasing concentrations of MMP-8 (10–1000 ng mL^−1^) measured in 0.1 M KCl containing 5 mM [Fe(CN)_6_]^3−^/^4−^. Distinct increases in semicircular diameter are observed for the MIP sensor, indicating concentration-dependent Z″ changes, whereas the NIP exhibits minimal variation. (**c**) Normalized ΔZ″ values as a function of MMP-8 concentration, illustrating the detection range and signal amplification of the MIP-based biosensor. (**d**) Cross-reactivity analysis between interference species, demonstrating relatively minor impedance changes for validating the molecular selectivity of the MMP-8 imprinted biosensor.

The proposed impedimetric analysis technique offers unique advantages, particularly in its ability to provide a comprehensive analysis of interfacial reaction characteristics and enable the evaluation of specific electrochemical processes occurring at the electrode surface. By performing frequency-dependent impedance response measurements, EIS independently characterizes critical parameters, including charge transfer resistance (*R*_ct_), double-layer capacitance (C_dl_), and diffusion processes. Thus, EIS can be beneficial in evaluating the molecular binding event between the MIP electrode and the target MMP-8 analyte because this analytical method is suited for detecting subtle changes at the electrode/electrolyte interface, even at low analyte concentrations, thereby enabling precise detection of MMP-8 rebinding into the imprinted cavities on the MIP electrode. In this study, the MMP-8 samples dispersed at various concentrations in buffer solution were introduced into the electrolyte, promoting the binding of MMP-8 to the imprinted cavities on the MIP electrode surface. As demonstrated in Figure 6a, the impedance response exhibited a concentration-dependent trend when MMP-8 solutions (ranging from 0 to 1000 ng mL^−1^) were exposed to the MIP electrode surface. EIS profiles were recorded at a potential of 0.1 V with an amplitude of 10 mV, logarithmically spanning frequencies from 0.1 to 100 kHz. An increase in the Nyquist plot’s semicircle diameter was observed with increased MMP-8 concentrations, which obviously reflects the progressive blocking of electron transfer (Figure S10a), caused by analyte recombination in the MMP-8–imprinted cavities. This phenomenon was supported by a corresponding increase in the −Z″ peak values with increasing MMP-8 concentration, signifying that the redox probe’s electron transfer was hindered due to the specific rebinding of MMP-8 within the imprinted cavities. Therefore, as shown in Figure S10b, this study shows that an increase in the Z″ value is indicative of a protein binding event, based on the following numerical equation (ΔZ″ ≈ Δ*R*_ct_/2). This simplified approach can reduce the analysis time by shortening the measurement time in the low-frequency analysis range. This simplified approach can reduce the analysis time by shortening the measurement time in the low-frequency analysis range on the basis of the excellent charge transport properties of the pEBT film, which is intimately connected to the underlying GO interlayer. In contrast to the MIP electrodes, the NIP electrode demonstrated negligible changes in Z″ values on the applied MMP-8 concentration ranges (Figure 6b), suggesting that the observed signal variations were predominantly due to nonspecific physical adsorption, which lacked molecular recognition capability. As presented in Figure 6c, quantitative analyses of the ΔZ″ values, where Z″ (blank) represents the baseline imaginary impedance after template extraction, revealed an approximately linear trend for the MIP-based sensing electrode across the concentration range (i.e., 0–1000 ng mL^−1^) with a correlation coefficient of [R^2^] = 0.9185. A visual change in slope is also evident around the mid-range, consistent with heterogeneous binding sites in protein-imprinted electrode (high-affinity sites at low concentrations vs. lower affinity/non-specific sites at higher concentrations) [71,75]. Conversely, the NIP electrode exhibited a low correlation coefficient (i.e., [*R*^2^] = 0.0744) with unstable responses, further emphasizing the MIP’s specificity and selectivity for MMP-8. The limit of detection (LOD) and limit of quantitation (LOQ) for the MIP sensor were determined using the equation k × S m^−1^, where S is the standard deviation (σ), m is the slope (i.e., sensitivity), and k is the signal-to-noise ratio [76]. The calculated values for LOD (k = 3) and LOQ (k = 10) were ~91.2 ng mL^−1^ and ~304 ng mL^−1^, respectively, demonstrating the practical applicability of the MIP-based biosensor for detecting MMP-8 at low concentrations [75,76]. Notably, EIS showed another aspect of sensitivity compared to SWV, as evidenced by the steeper response slope (m = 0.097) in the concentration range of 0–1000 ng mL^−1^, compared to SWV’s slope (m = 0.006) over the same range. This indicates the enhanced ability of EIS to capture fine concentration-dependent changes in molecular interactions [71]. Compared with recent reports (Table S3), our MIP-based biosensor exhibited a lower LOD that remains within the pathological range for periodontitis and a wider dynamic range.

To further assess the selectivity of the MIP-based biosensor, interference studies were conducted using potential interferent proteins, including IL-1β, TNF-α, Myo, and IgG. Figure 6d presents the EIS responses for these proteins, all tested at an identical concentration (i.e., 1000 ng mL^−1^). Each protein exhibited distinct levels of ΔZ″ increase, highlighting their varying degrees of interaction with the MMP-8-imprinted MIP electrode. The individual Z″ values for IL-1β and TNF-α displayed relatively low increases (~20–22%) compared to the significant response observed for MMP-8 (~110%), representing the excellent selectivity of the sensor toward the target biomarker. In contrast, Myo and IgG demonstrated slightly higher increases (~30–40%) with a minor degree of interference by the rebinding process. Such relatively high interference can be derived from the charge states of interfering proteins, which can affect the binding affinity of the sensing electrode due to molecular interactions (e.g., isoelectric point effects) even in an environment designed to detect the specific biomarker MMP-8. Nevertheless, the Z″ levels for the interfering proteins were apparently lower than those recorded for MMP-8 and MMP-8/interferents, which validates the MIP electrode’s selective recognition capability. Furthermore, the MIP electrode exhibited clear concentration-dependent responses despite the influence of multiple interfering species. These experimental results denote that the charge or isoelectric point differences in the interferents might cause minor baseline shifts but do not hinder MMP-8 binding significantly. While various factors may disrupt the rebinding of MMP-8 at the cavity sites on the MIP electrode, the results of these selectivity experiments affirm the presented biosensor’s ability to accurately detect MMP-8 over a defined concentration range, highlighting its potential utility as a diagnostic tool. Despite these promising results, optimizing the complete molecular capture efficiency of the MIP-based biosensor remains an area requiring further investigation. In particular, the development of a standardized interference evaluation protocol is essential to verify its selective detection performance, especially in a clinically relevant sample matrix. For instance, testing the selective detection of MMP-8 in human salivary aqueous solutions, where multiple interfering protein species coexist, is a critical step toward dental clinical applications. To mitigate crosstalk and enable multiplexed analysis, advances in surface-imprinting architectures are also needed. These could include spatially segregated multi-template microarrays, orthogonal binding sites, and anti-fouling interfaces on electrochemically addressable electrodes. Rationally designed MIP architectures along these lines could extend the MIP-based biosensor platform towards multi-analyte dental diagnostics, which is an active area of research within the community.

## 3. Conclusions

In summary, we developed a MIP-based electrochemical biosensor for the selective and sensitive detection of MMP-8, employing a stepwise electropolymerization strategy on SPCEs. By introducing a conformal layer of rGO onto the electrode surface, we engineered a conductive and chemically active interface that facilitated the subsequent polymer growth and imprinting process. The EBT monomer and MMP-8 template were co-assembled onto this rGO-modified surface, yielding an artificial receptor matrix with nanoscale molecular recognition cavities formed through template extraction. Surface characterizations using AFM and XPS confirmed the successful deposition of the PEBT layer and the creation of well-defined imprinted sites. Notably, nitrogen spectral deconvolution revealed the selective removal of protein-associated functional groups, verifying the molecular-level fidelity of the imprinting process. Electrochemical analyses further demonstrate consistent and concentration-dependent signal responses toward MMP-8, while control experiments with structurally related proteins validated the selectivity of the sensor. Complementary DFT calculations provided atomistic insights into the interaction background between EBT and MMP-8, revealing binding energy preferences within the catalytic domain. These findings reinforce the hypothesis that pre-polymerization complexation is a key determinant of recognition site specificity in MIP-based sensors. Additionally, fabrication parameters, including solvent composition and extraction cycles, were systematically optimized to ensure stable sensor performance and reproducibility. The final sensor design exhibited reliable signal saturation within 50 min, supporting its suitability for real-time bioanalytical applications, biomarkers in complex biological matrices such as saliva.

## 4. Experimental Section

### 4.1. Materials

Eriochrome black T (EBT), potassium hexacyanoferrate (K_3_[Fe(CN)_6_]), potassium hexacyanoferrate trihydrate (K_4_[Fe(CN)_6_]·3H_2_O), phosphate-buffered saline (PBS, 0.01 M; pH: 7.4), phosphate-buffered solution (PB, 0.1 M, pH: 7.0), Tris buffer solution (pH = 8.5, 1 M; BTH-9185), Acetonitrile (99.8%), Lithium perchlorate (LiClO_4_, >95%), myoglobin (Myo), immunoglobulin G (IgG) were purchased from Sigma-Aldrich Co. (St. Louis, MO, USA). Matrix metalloproteinase-8 (MMP-8) (MW = 44.3 kDa) and Interleukin-1β (IL-1β) (MW = 17.4 kDa) were purchased from NKMAX Co. Ltd. (Seongnam, Republic of Korea). Graphene Oxide solution was purchased from Graphene Supermarket Inc. (Calverton, NY, USA).

### 4.2. MIP-Based Biosensor Fabrication

MIP-based biosensors were fabricated on commercial screen-printed carbon electrodes (SPCE, C11L, Metrohm DropSens, Oviedo, Spain) by electropolymerization using a potentiostat (CompactStat, Ivium Technologies BV, Eindhoven, The Netherlands). First, the SPCE surface was electrochemically activated by cyclic voltammetry (CV) in 0.01 M phosphate-buffered saline (PBS), scanning from −1.05 to +0.65 V at 50 mV s^−1^ for 10 cycles. After activation, electrochemical reduction–deposition of graphene oxide (GO) onto the electrode was carried out in an ionic medium (0.067 mM PBS, 0.1 M LiClO_4_). This step was performed by CV between −1.50 and +0.60 V at 50 mV s^−1^ for 5 cycles. Subsequently, a thin PEBT film was formed on the rGO-modified SPCE in the presence of the MMP-8 template. A polymerization solution prepared in 0.1 M phosphate buffer (PB) containing 0.2 M acetonitrile (ACN), 0.58 × 10^−6^ M MMP-8, and 1.0 × 10^−3^ M eriochrome black T (EBT) was used; CV was applied from −0.45 to +0.90 V at 100 mV s^−1^ for 8 cycles. The resulting PEBT-based MIP electrodes were rinsed with deionized water and dried under nitrogen. To generate molecular recognition cavities, the template molecules were removed by performing 25 CV cycles in 0.2 M ACN over −0.80 to +0.20 V at 100 mV s^−1^. Non-imprinted polymer (NIP) electrodes were prepared under identical conditions, except that the MMP-8 template was omitted.

### 4.3. Surface Characterization

The surface morphology and topography of the SPCEs at each deposition step were characterized using field-emission scanning electron microscopy (FE-SEM) (SUPRA 25 VP, Carl Zeiss SMT, Oberkochen, Germany) and atomic force microscopy (AFM) (XE-100, Park Systems, Suwon, Republic of Korea) operating in non-contact mode. The electrochemically reduced GO films were analyzed via Raman spectroscopy (UniNanoTech, UniRam-II, Yong-in, Republic of Korea) using 532 nm laser excitation across a wavelength range of 500–3500 nm. Raman measurements were conducted with an acquisition time of 10 s and accumulated three times for a total duration of 30 s. The chemical composition at each deposition step was examined using X-ray photoelectron spectroscopy (XPS) (AXIS Supra, Kratos Analytical Ltd., Manchester, UK) equipped with an Al-Kα excitation source (1486.6 eV).

### 4.4. Characterization of the MIP Sensor Performance

All electrochemical measurements of the MIP- and NIP-based electrodes for MMP-8 sensing were performed using a potentiostat (CompactStat, Ivium Technologies BV, Eindhoven, The Netherlands) in 0.1 M KCl (pH 6.0) containing 5 mM K_3_[Fe(CN)_6_]/K_4_[Fe(CN)_6_] redox probes. Prior to measurements, the working electrode was exposed to 0.5 M Tris-HCl buffer for 10 min to establish baseline signals. Square wave voltammetry (SWV) was conducted in a potential range of −0.2 to +0.5 V at a frequency of 25 Hz with a 25 mV pulse amplitude and 10 mV step potential. EIS measurements were recorded under the 0.1 Hz–100 kHz frequency range at 0.1 V potential and 10 mV amplitude. The EIS data analysis was based on the peak value of the imaginary impedance obtained from the Nyquist plot. To evaluate the binding characteristics of the MMP-8-imprinted MIP films, EIS responses were recorded after 60-min incubation with MMP-8 standard solutions (0–1000 ng mL^−1^ in 0.5 M Tris-HCl). The specific binding of the MMP-8-imprinted MIP films was investigated using solution samples of MMP-8, IL-1β, IgG, and Myo at a concentration of 1000 ng mL^−1^.

### 4.5. Data Analysis and Statistics

All experimental measurements were performed in triplicate using the MIP-based sensor, and the results are expressed as mean values with their corresponding standard deviations (mean ± SD). The significance level was set at *p* < 0.05 for statistical analysis. Sensor reproducibility was evaluated using the relative standard deviation of measurements. The analytical performance parameters, including the LOD and LOQ, were calculated using the standard formulas: LOD = 3.3 × (SD/slope) and LOQ = 10 × (SD/slope), based on the calibration curve analysis. Data processing and statistical analyses were conducted using OriginLab version 8.0.

### 4.6. Simulation of Molecular Electrostatic Potential

To estimate the binding energy of the molecular imprinting complex, the ESP of the pre-polymerization complex was calculated using DFT. The ground-state electronic energies of the molecular systems were computed using the B3LYP functional with the 6-31+G basis set, as implemented in the Gaussian 16W software package (Gaussian, Inc., Wallingford, CT, USA). For molecular structure optimization, GaussView 6.0.1 (Semichem Inc., Shawnee Mission, KS, USA) was employed to construct the spatial configurations of EBT and representative amino acids, including alanine, which are constituent residues of the MMP-8 protein sequence. The geometries of these amino acid–EBT complexes were initially optimized using the semi-empirical PM6 method. To mimic the neutrality and truncation of the peptide chain, terminal groups of the amino acid residues were hydrogen-capped. Subsequently, electrostatic potential maps for all 20 amino acids were generated to assess interaction profiles across the full MMP-8 sequence. The complete protein sequence and its three-dimensional structure were obtained from the UniProt/PDB database under the accession code P22894 (AlphaFold prediction by Google DeepMind, London, UK). ESP surfaces were mapped onto the van der Waals surface of the MMP-8 protein using the SAMSON Connect molecular modeling platform. This approach enabled a visual and quantitative comparison of the ESP distribution across the protein surface, thereby identifying regions of favorable interaction and potential imprinting sites for the EBT monomer within the catalytic and auxiliary domains of MMP-8.

## Data Availability

Data are contained within the article or Supplementary Materials.

## Supplementary Materials

The following supporting information can be downloaded at: https://www.mdpi.com/article/10.3390/bios15100671/s1, Figure S1: High-resolution XPS survey spectra of rGO-modified electrode; (a) C 1s, (b) O 1s and (c) N 1s spectra. Figure S2: (a) EIS and (b) SWV responses for SPCE and rGO-modified SPCE. Figure S3: SEM image representing the surface of NIP-modified rGO/SPCE. Figure S4: EIS responses of stepwise-modified NIP electrodes. Figure S5: Stepwise evaluated SWV response of the NIP electrode; Figure S6: pH-dependent sensory response for 2-step optimization process by electrochemical characterization. The sensory responses were normalized to numerical values by calculating the ratio of the imaginary impedance changes in the NIP and MIP electrodes, using the following equation: Δ(Z) = Δ(Z″)_MIP_/Δ(Z″)_NIP_; (a,b) Rebinding step performed in buffer solutions of pH 6.0 (0.1 M KCl), pH 7.4 (0.01 M PBS), and pH 8.0 (0.5 M Tris–HCl). (c–d) Electrochemical readout carried out in 0.1 M KCl, 0.01 M PBS and 0.5 M Tris-HCl, with 5 mM [Fe(CN)_6_]^3−^/^4−^ redox probes. Larger Δ(Z″) values indicate stronger MIP-specific binding capability. Figure S7: High-resolution XPS survey spectra of NIP-modified electrode (NIP/rGO/SPCE); (a) C 1s, (b) O 1s and (c) N 1s spectra. Figure S8: Structural segmentation of MMP-8 protein and domain-specific electrostatic analysis. The MMP-8 structure was modeled using an AlphaFold-predicted structural model (UniProt: P22894, Google DeepMind, London, UK), including the pre-domain region (residues 1–108). (a) Electrostatic surface map of MMP-8, highlighting the catalytic domain (residues 109–275, red dashed line) and the propeptide domain (residues 1–108, purple dashed line); the inset depicts the active MMP-8, based on its crystal structure. (b) DFT-derived, sequence-resolved binding energy profile of local EBT–amino acid interactions along the 467-residue MMP-8 chain. Figure S9: Temporal response characteristics of the optimized sensor at a fixed MMP-8 concentration (n = 3). Figure S10: (a) Schematic illustration of impedimetric sensing mechanism with redox probe ions. (b) Equivalent circuit model used for quantitative interpretation of selective rebinding of MMP-8. Table S1: Elemental composition (at. %) of the rGO/SPCE and the MIP/rGO/SPCE (before and after MMP-8 extraction) extracted from the XPS analysis. Table S2: Hydrogen bond parameters between the EBT and the 20 amino acid molecules. Table S3: Analytical performances of different biosensors for MMP-8 detection. Supplementary Video S1: Computational simulation: The formation of EBT/MMP-8 prepolymerization complex. References [77,78,79,80] are cited in the Supplementary Materials.
