# Supplementary material for: Rationally Designed Molecularly Imprinted Polymer Electrochemical Biosensor with Graphene Oxide Interface for Selective Detection of Matrix Metalloproteinase-8 (MMP-8)
Source: Biosensors (Basel). 2025 Oct 4;15(10):671. doi: 10.3390/bios15100671 (PMC12562401; doi:10.3390/bios15100671)
Supplement: Supplementary file 1 [file biosensors-15-00671-s001.zip › Supplementary Materials.pdf]

Rationally Designed Molecularly Imprinted Polymer Electrochemical Biosensor with Graphene Oxide Interface for Selective Detection of Matrix Metalloproteinase-8 (MMP-8)

Jae Won Lee [1,†], Rowoon Park [1,†], Sangheon Jeon [1], Sung Hyun Kim [2], Young Woo Kwon [2], Dong-Wook Han [1] and Suck Won Hong [1,*]

[1] Department of Cogno-Mechatronics Engineering, College of Nanoscience and Nanotechnology, Pusan National University, Busan 46241, Republic of Korea; jaewon03135@gmail.com (J.W.L.); rowoon.p153@gmail.com (R.P.); sangheon.jn@gmail.com (S.J.); nanohan@pusan.ac.kr (D.-W.H.)

[2] Engineering Research Center for Color-Modulated Extra-Sensory Perception Technology, Pusan National University, Busan 46241, Republic of Korea; ksh1332@pusan.ac.kr (S.H.K.); ywkwon@pusan.ac.kr (Y.W.K.)

* Correspondence: swhong@pusan.ac.kr, TEL: 82-51-510-6119

† These authors contributed equally to this work.

KEYWORDS: molecularly imprinted polymers, matrix metalloproteinase-8, cytokine, electrochemical impedance spectroscopy, point-of-care testing

Table S1. Elemental composition (at. %) of the rGO/SPCE and the MIP/rGO/SPCE (before and after MMP-8 extraction) extracted from the XPS analysis.

Element	Atomic concentration (%)		
	C 1s	O 1s	N 1s
rGO/SPCE	82.52	14.87	2.61
MIP/rGO/SPCE (with MMP-8)	77.6	17.5	4.12
MIP/rGO/SPCE (MMP-8 removal)	81.51	15.7	2.15

Table S2. Hydrogen bond parameters between the EBT and the 20 amino acid molecules.

Molecule structure	Energy of the spatial structure (Hartree)	Binding energy of amino acid-EBT (kJ mol^{-1})	Hydrogen bond Length (Å)	Mulliken charge of targeting Oxygen (δ)
**EBT**	**-1857.420101**	**free monomer**		
Alanine (Ala)	-248.422666	Template amino acid		
EBT-Ala (1)	-2105.837524	13.765	1.89	-0.420
EBT-Ala (2)	-2105.833687	23.840	1.95	-0.578
EBT-Ala (3)	-2105.813399	77.106	2.32	-0.655
EBT-Ala (4)	-2105.830806	31.404	1.55	-0.333
**1. EBT-Ala total**		**146.115**		
Arginine (Arg)	-531.593043	Template amino acid	-	-
EBT-Arg (1)	-2389.006119	18.444	2.44	-0.664
EBT-Arg (2)	-2389.004026	23.940	2.47	-0.431
EBT-Arg (3)	-2388.980147	86.633	2.99	-0.607
EBT-Arg (4)	-2388.984379	75.523	3.24	-0.341
**2. EBT-Arg total**		**204.54**		
Asparagine (Asn)	-417.051539	Template amino acid		
EBT-Asn (1)	-2274.468174	9.100	2.49	-0.473
EBT-Asn (2)	-2274.457040	38.333	2.54	-0.451
EBT-Asn (3)	-2274.449125	59.113	2.67	-0.626
EBT-Asn (4)	-2274.441440	79.291	2.33	-0.280
**3. EBT-Asn total**		**185.837**		
Aspartic Acid (Asp)	-436.346837	Template amino acid	-	-
EBT-Asp (1)	-2293.759472	19.602	2.03	-0.462
EBT-Asp (2)	-2293.736226	53.798	2.33	-0.527
EBT-Asp (3)	-2293.740248	80.635	2.62	-0.640
EBT-Asp (4)	-2293.746447	70.075	2.80	-0.285
**4. EBT-Asp total**		**224.110**		
Cysteine (Cys)	-284.0012695	Template amino acid		
EBT-Cys (1)	-2141.4139045	23.333	1.94	-0.410
EBT-Cys (2)	-2141.3906585	23.444	2.01	-0.518
EBT-Cys (3)	-2141.3946805	77.121	2.22	-0.644
EBT-Cys (4)	-2141.4008795	61.211	1.88	-0.323
**5. EBT-Cys total**		**185.109**		
Glutamine (Gln)	-456.345712	Template amino acid	-	-
EBT-Gln (1)	-2313.763244	**6.745**	2.38	-0.483
EBT-Gln (2)	-2313.734143	83.149	2.20	-0.597
EBT-Gln (3)	-2313.722483	113.764	2.14	-0.642
EBT-Gln (4)	-2313.754975	28.455	2.58	-0.289
**6. EBT-Gln total**		**232.113**		

Glutamic Acid (Glu)	-475.643492	Template amino acid	-	-
EBT-Glu (1)	-2333.058907	12.302	2.35	-0.526
EBT-Glu (2)	-2333.051405	32.000	2.57	-0.442
EBT-Glu (3)	-2333.039015	64.530	2.77	-0.590
EBT-Glu (4)	-2333.046408	45.121	2.31	-0.321
**7. EBT-Glu total**		**153.953**		
Glycine (Gly)	-209.100112	Template amino acid		
EBT-Gly (1)	-2066.510703	24.969	2.08	-0.408
EBT-Gly (2)	-2066.519264	2.492	2.13	-0.577
EBT-Gly (3)	-2066.512807	19.444	2.54	-0.630
EBT-Gly (4)	-2066.513079	18.730	2.21	-0.302
**8. EBT-Gly total**		**65.635**		
Phenylalanine (His)	-309.9162961	Template amino acid	-	-
EBT-His (1)	-2167.331154	25.321	2.11	-0.401
EBT-His (2)	-2167.327317	21.851	2.31	-0.540
EBT-His (3)	-2167.307029	55.234	3.32	-0.600
EBT-His (4)	-2167.324436	44.601	4.20	-0.301
**9. EBT-His total**		**147.007**		
Isoleucine (Ile)	-365.7579996	Template amino acid	-	-
EBT-Ile (1)	-2223.172858	15.3217	2.29	-0.410
EBT-Ile (2)	-2167.327317	13.844	2.87	-0.520
EBT-Ile (3)	-2167.307029	45.221	3.19	-0.588
EBT-Ile (4)	-2167.324436	33.221	4.29	-0.312
**10. EBT-Ile total**		**107.607**		
Leucine (Leu)	-366.306240	Template amino acid		
EBT-Leu (1)	-2223.718982	19.321	4.54	-0.637
EBT-Leu (2)	-2223.721631	12.365	4.31	-0.451
EBT-Leu (3)	-2223.725918	1.111	4.92	-0.632
EBT-Leu (4)	-2223.726051	0.761	5.40	-0.343
**11. EBT-Leu total**		**33.558**		
Lysine (Lys)	-422.096607	Template amino acid		
EBT-Lys (1)	-2279.493463	61.030	2.03	-0.625
EBT-Lys (2)	-2279.507590	23.940	2.15	-0.579
EBT-Lys (3)	-2279.491325	66.643	2.58	-0.629
EBT-Lys (4)	-2279.510807	15.493	2.15	-0.400
**12. EBT-Lys total**		**167.106**		
Methionine (Met)	-725.157334	Template amino acid	-	-
EBT-Met (1)	-2582.567942	24.924	2.63	-0.643
EBT-Met (2)	-2582.564610	33.672	2.65	-0.598
EBT-Met (3)	-2582.564858	33.021	2.65	-0.632
EBT-Met (4)	-2582.571693	15.076	2.43	-0.301
**13. EBT-Met total**		**106.693**		

Phenylalanine (Phe)	-479.383724	Template amino acid	-	-
EBT-Phe (1)	-2336.794185	25.310	4.32	-0.621
EBT-Phe (2)	-2336.803707	0.310	2.33	-0.440
EBT-Phe (3)	-2336.803673	0.401	5.62	-0.600
EBT-Phe (4)	-2336.803558	0.699	6.80	-0.301
**14. EBT-Phe total**		**26.72**		
Proline (Pro)	-325.795736	Template amino acid	-	-
EBT-Pro (1)	-2183.206075	25.630	2.25	-0.393
EBT-Pro (2)	-2183.215328	1.337	2.84	-0.583
EBT-Pro (3)	-2183.215603	0.615	2.22	-0.640
EBT-Pro (4)	-2183.212880	7.764	2.39	-0.311
**15. EBT-Pro total**		**35.345**		
Serine (Ser)	-323.584919	Template amino acid	-	-
EBT-Ser (1)	-2180.988028	44.612	2.42	-0.492
EBT-Ser (2)	-2180.982213	59.880	2.34	-0.617
EBT-Ser (3)	-2180.992089	33.950	2.44	-0.614
EBT-Ser (4)	-2181.003416	4.211	2.21	-0.305
**16. EBT-Ser total**		**142.653**		
Threonine (Thr)	-362.886784	Template amino acid	-	-
EBT-Thr (1)	-2220.300411	16.997	2.13	-0.669
EBT-Thr (2)	-2220.305235	4.332	2.76	-0.576
EBT-Thr (3)	-2220.294442	32.669	2.81	-0.642
EBT-Thr (4)	-2220.306882	0.008	2.66	-0.297
**17. EBT-Thr total**		**54.006**		
Tryptophan (Trp)	-569.4559976	Template amino acid		
EBT-Trp (1)	-2426.870856	11.565	2.51	-0.410
EBT-Trp (2)	-2426.867019	25.976	2.25	-0.532
EBT-Trp (3)	-2426.846731	67.235	2.55	-0.578
EBT-Trp (4)	-2426.864138	42.555	2.00	-0.400
**18. EBT-Trp total**		**147.331**		
Tyrosine (Tyr)	-554.556305	Template amino acid		
EBT-Tyr (1)	-2411.964316	31.742	2.31	-0.649
EBT-Tyr (2)	-2411.957141	50.580	2.48	-0.586
EBT-Tyr (3)	-2411.965785	27.885	2.72	-0.639
EBT-Tyr (4)	-2411.962481	36.561	2.52	-0.284
**19. EBT-Tyr total**		**146.768**		
Valine (Val)	-323.216119	Template amino acid		
EBT-Val (1)	-2180.629608	17.361	3.11	-0.661
EBT-Val (2)	-2180.630982	13.752	2.87	-0.451
EBT-Val (3)	-2180.628172	21.131	3.07	-0.636
EBT-Val (4)	-2180.626796	24.744	3.42	-0.330
**20. EBT-Val total**		**76.988**		

Table S3. Analytical performances of different biosensors for MMP-8 detection. Despite of our sensor showed higher LOD than previous reports, it showed notable detection range than previous reports, especially for antibody-based sensor platforms.

Platform/Sensor type	Recognition	Transduction	Electrode /Substrate	Detection range (ng mL^{-1})	LOD (ng mL^{-1})	Sample matrix	Ref.
This work (rGO/pEBT MIP) @ Electrochemical	MIP	SWV/EIS	GO-modified @MIP layer	0-1000	91.2	Buffer	—
rGO/o-PD MIP @ Electrochemical	MIP	EIS	GO-modified @MIP layer	0-500	82	Buffer, Saliva	[1]
Au nanosphere/Antibody @ ELISA	Antibody	DPV	Au nanosphere @graphene	1-50	4.12	Saliva (vs ELISA)	[2]
MUA/Au/Antibody @ ELISA	Antibody	DPV	Au SPE @MUA	2.5-300	1.0 ± 0.1	Buffer (vs ELISA)	[3]
Dual-channel electrochemical immunosensor	Antibody	DPV (microchip)	IrO_2 nanotubes @Mxene	0-200	2.15	Artificial /Clinical Saliva	[4]
SAW biosensor (SPR-POF)	Antibody	Piezoelectric biochip	Fiber probe	—	9.9	Saliva	[5]

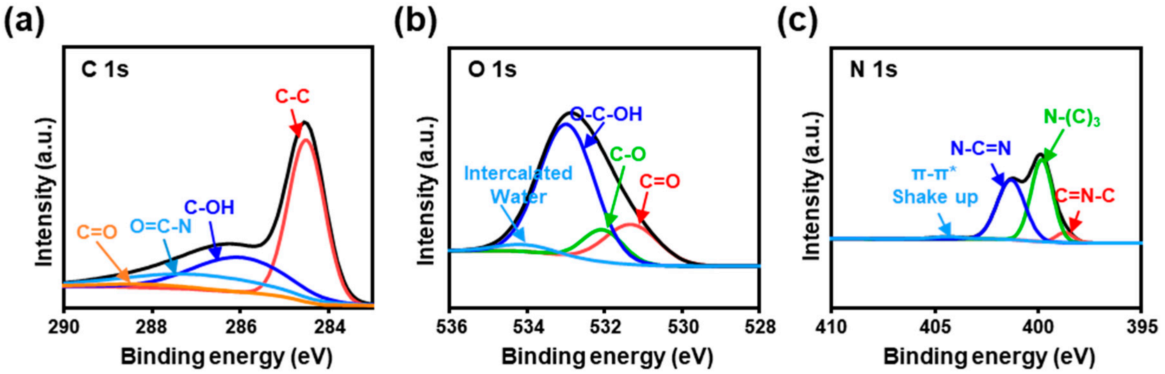

Figure S1. High-resolution XPS survey spectra of rGO-modified electrode; (a) C 1s, (b) O 1s and (c) N 1s spectra.

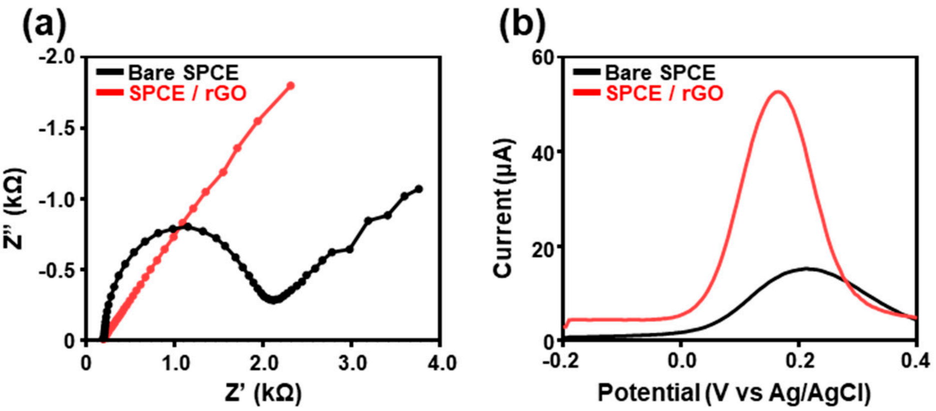

Figure S2. (a) EIS and (b) SWV responses for SPCE and rGO-modified SPCE; all measurements were performed in 0.1 M KCl with a 5 mM $[Fe(CN)_6]^{3-/4-}$ redox solution.

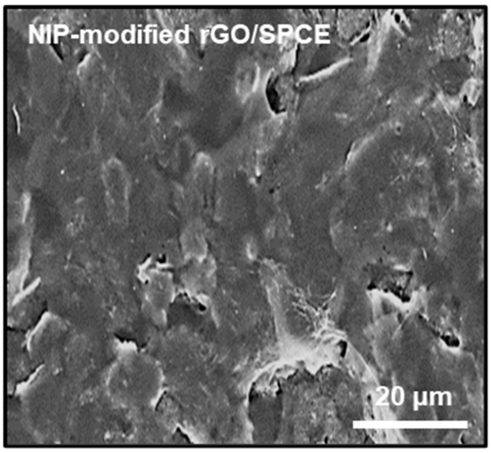

Figure S3. SEM image of NIP-modified rGO/SPCE.

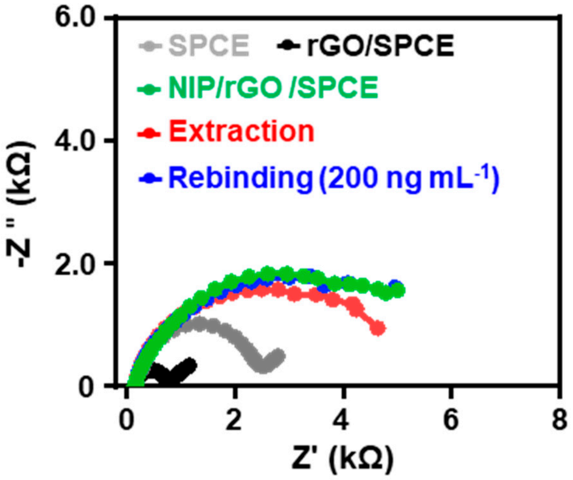

Figure S4. EIS responses of stepwise-modified NIP electrodes; all measurement was conducted in 0.1 M KCl solution containing 5 mM $[Fe(CN)_6]^{3-/4-}$ as the redox probe.

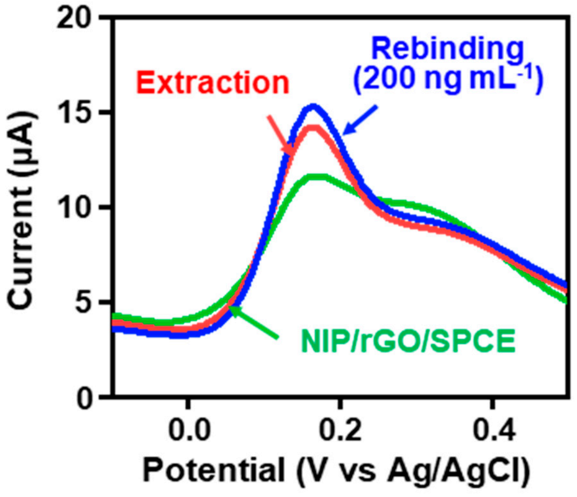

Figure S5. Stepwise evaluated SWV response of the NIP electrode; all measurements were performed in 0.1 M KCl with a 5 mM $[Fe(CN)_6]^{3-/4-}$ redox solution.

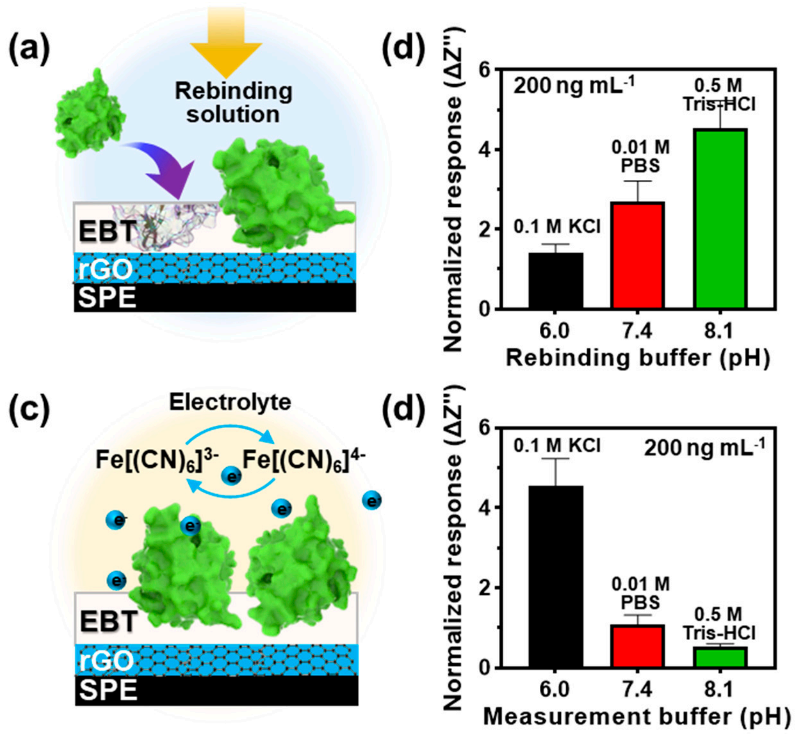

Figure S6. pH-dependent sensory response during the two-step optimization process based on electrochemical characterization. The sensory responses were normalized to numerical values by calculating the ratio of the imaginary impedance changes in the NIP and MIP electrodes, using the following equation: $\Delta(Z) = \Delta(Z'')_{MIP}/\Delta(Z'')_{NIP}$; (a-b) Rebinding step performed in buffer solutions of pH 6.0 (0.1 M KCl), pH 7.4 (0.01 M PBS), and pH 8.0 (0.5 M Tris–HCl). (c-d) Electrochemical readout carried out in 0.1 M KCl, 0.01 M PBS and 0.5 M Tris-HCl, with 5 mM $[Fe(CN)_6]^{3-/4-}$ redox probes. Larger Δ(Z") values indicate stronger MIP-specific binding capability.

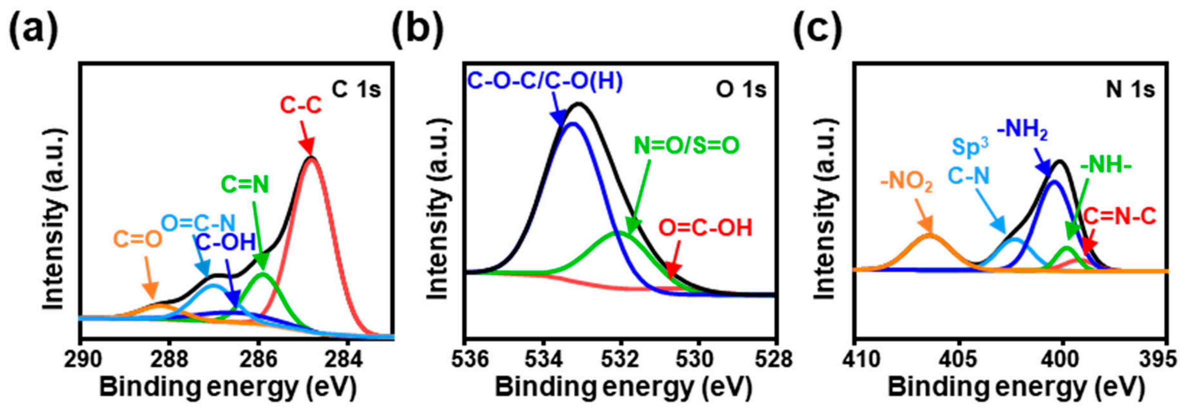

Figure S7. High-resolution XPS survey spectra of NIP-modified electrode (NIP/rGO/SPCE); (a) C 1s, (b) O 1s and (c) N 1s spectra.

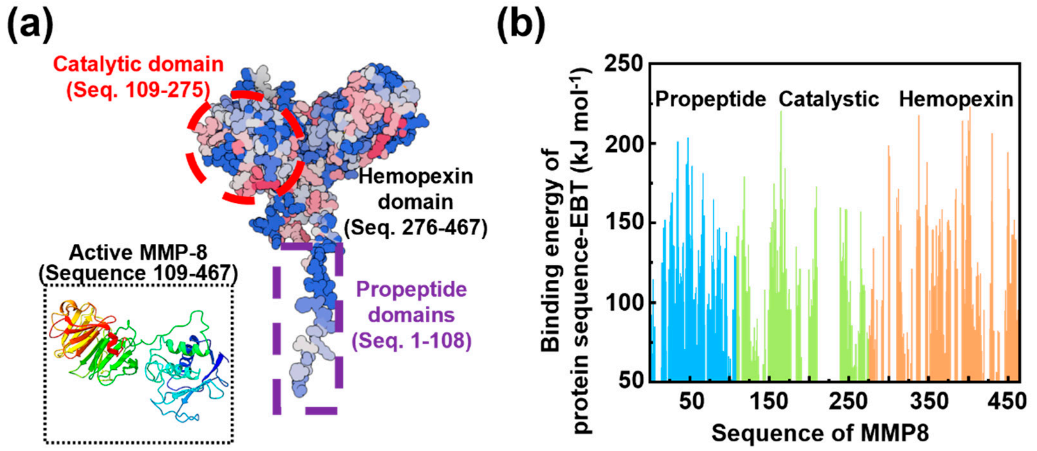

Figure S8. Structural segmentation of MMP-8 protein and domain-specific electrostatic analysis. The MMP-8 structure was modeled using an AlphaFold-predicted structural model (UniProt: P22894, Google DeepMind, USA), including the pre-domain region (residues 1–108). (a) Electrostatic surface map of MMP-8, highlighting the catalytic domain (residues 109–275, red dashed line) and the propeptide domain (residues 1–108, purple dashed line); the inset depicts the active MMP-8, based on its crystal structure. (b) DFT-derived, sequence-resolved binding energy profile of local EBT–amino acid interactions along the 467-residue MMP-8 chain.

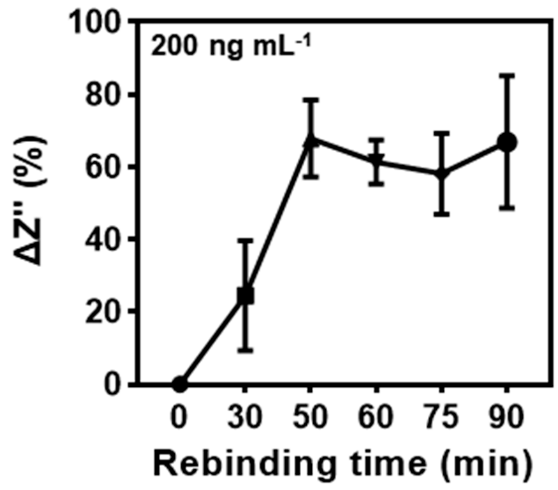

Figure S9. Temporal response characteristics of the optimized sensor at a fixed MMP-8 concentration (n = 3).

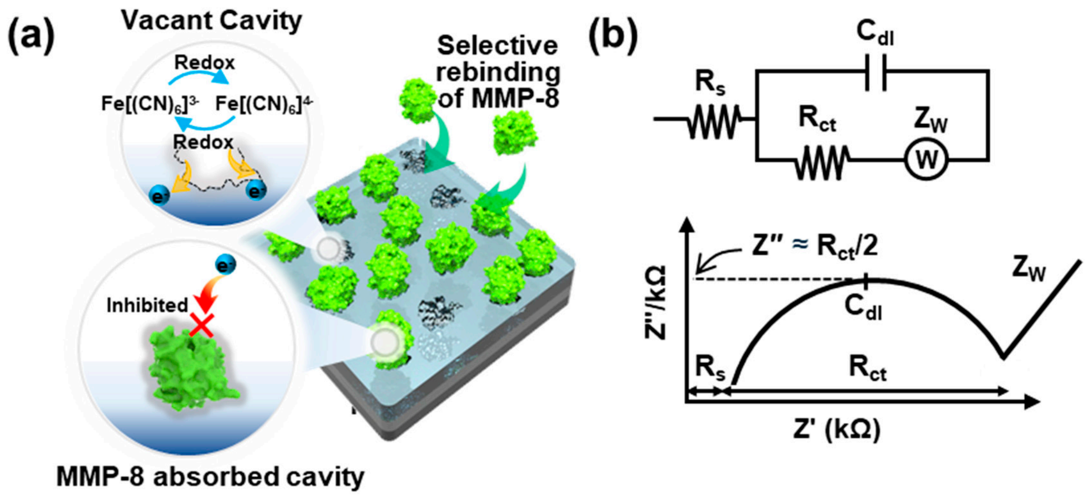

Figure S10. (a) Schematic illustration of impedimetric sensing mechanism with redox probe ions. (b) Equivalent circuit model used for quantitative interpretation of selective rebinding of MMP-8.

References

1. 1. Jeon, S.; Kim, S.H.; Heo, G.; Heo, H.J.; Chae, S.Y.; Kwon, Y.W.; Lee, S.-K.; Han, D.-W.; Kim, H.-J.; Kim, Y.H.; et al. A Wearable Electrochemical Biosensor for Salivary Detection of Periodontal Inflammation Biomarkers: Molecularly Imprinted Polymer Sensor with Deep Learning Integration. *Adv. Sci.* **2025**, e09658. DOI: 10.1002/advs.202509658
2. 2. Tortolini, C.; Gigli, V.; Angeloni, A.; Tasca, F.; Thanh, N.T.K.; Antiochia, R. A Disposable Immunosensor for the Detection of Salivary MMP-8 as Biomarker of Periodontitis. *Bioelechem.* **2024**, *156*, 108590. DOI: 10.1016/j.bioelechem.2023.108590
3. 3. Lowpradit, P.; Janmanee, R.; Tansriratanawong, K**.** Performance Validation of Fabricated Nanomaterial-Based Biosensor for Matrix Metalloproteinase-8 Protein Detection. *European Journal of Dentistry* **2025**. DOI: 10.1055/s-0045-1809182
4. 4. Zhang, W.; Du, J.; Wang, K.; Li, Y.; Chen, C.; Yang, L.; Kan, Z.; Dong, B.; Wang, L.; Xu, L. Integrated Dual-Channel Electrochemical Immunosensor for Early Diagnosis and Monitoring of Periodontitis by Detecting Multiple Biomarkers in Saliva. *Analytica Chimica Acta* **2023**, *1247*, 340878. DOI: 10.1016/j.aca.2023.340878
5. 5. Guida, L.; Bencivenga, D.; Annunziata, M.; Arcadio, F.; Borriello, A.; Della Ragione, F.; Formisano, A.; Piccirillo, A.; Zeni, L.; Cennamo, N. An Optical Fiber-Based Point-of-Care Test for Periodontal MMP-8 Detection: A Proof of Concept. *Journal of Dentistry* **2023**, 134, 104553.DOI: 10.1016/j.jdent.2023.104553
